# Cannabinoid hyperemesis syndrome: genetic susceptibility to toxic exposure

**DOI:** 10.3389/ftox.2024.1465728

**Published:** 2024-10-23

**Authors:** Ethan B. Russo, Venetia L. Whiteley

**Affiliations:** CReDO Science, Austin, TX, United States

**Keywords:** cannabinoid hyperemesis syndrome, cannabinoids, tetrahydrocannabinol, cannabis, nausea, vomiting, abdominal pain, genomics

## Abstract

Cannabinoid hyperemesis syndrome presents as a complex of symptoms and signs encompassing nausea, vomiting, abdominal pain, and hot water bathing behavior, most typically in a heavy cannabis user. Its presentation is frequently associated with hypothalamic-pituitary-adrenal axis activation with stress and weight loss. Recent investigation has identified five statistically significant mutations in patients distinct from those of frequent cannabis users who lack the symptoms, affecting the TRPV1 receptor, two dopamine genes, the cytochrome P450 2C9 enzyme that metabolizes tetrahydrocannabinol, and the adenosine triphosphate-binding cassette transporter. The syndrome is associated with escalating intake of high potency cannabis, or alternatively, other agonists of the cannabinoid-1 receptor including synthetic cannabinoids. Some patients develop environmental triggers in scents or foods that suggest classical conditioned responses. Various alternative “causes” are addressed and refuted in the text, including exposure to pesticides, neem oil or azadirachtin. Nosological confusion of cannabinoid hyperemesis syndrome has arisen with cyclic vomiting syndrome, whose presentation and pathophysiology are clearly distinct. The possible utilization of non-intoxicating antiemetic cannabis components in cannabis for treatment of cannabinoid hyperemesis syndrome is addressed, along with future research suggestions in relation to its genetic foundation and possible metabolomic signatures.

## 1 Introduction

Cannabinoid hyperemesis syndrome (CHS) is an enigmatic constellation of symptoms and signs first reported in Australia in 2004, but with an index case beginning in 1996 with an 8-year gap before identification (Allen et al., 2004). Cases are remarkably stereotyped in their presentation in the intervening 20 years and are marked by features of subacute to chronic episodes of nausea, vomiting, abdominal pain, and hot water bathing behavior that are associated with heavy cannabis usage (DeVuono and Parker, 2020) or synthetic agonists of the CB_1_ receptor, such as JWH compounds (*vide infra*) (Hopkins and Gilchrist, 2013) and delta-8-tetrahydrocannabinol (Rosenthal et al., 2021). Whereas tetrahydrocannabinol (THC), the primary psychoactive component of *Cannabis sativa* and a partial agonist at the CB_1_ receptor, and herbal cannabis are recognized as anti-emetic in small doses, they are subject to biphasic dose responses, such that any person, irrespective of tolerance factors, may experience isolated nausea and vomiting when an excessive dose is inhaled or ingested. One-time misadventures must then be distinguished from the more prolonged time course of CHS that may present in phases (DeVuono and Parker, 2020), consisting of a prodrome of anxiety and diaphoresis accompanying nausea and vomiting, followed by protracted severe nausea, vomiting, abdominal pain with hot water bathing or showers temporarily alleviating symptoms. Acute intervention with intravenous administration of haloperidol, a dopamine D2 agonist (after typical failure of response to serotonin type 3 receptor antagonists) and/or cutaneous application of capsaicin (a TRPV1 agonist along with ethanol and heat stimuli) may allow a third phase of slow diminution and disappearance of symptoms, but only if the patient remains abstinent from herbal cannabis, products containing THC, or other CB1 agonists, whether natural or synthetic. The requirement for abstinence to eliminate typical symptoms was noted by in the first report on CHS (Allen et al., 2004), and remains the therapeutic recommendation to the current time (DeVuono and Parker, 2020). Most commonly, the syndrome appears in heavy cannabis users. In the largest study to date, survey data reveal that 89% of 205 confirmed CHS patients utilized an average of 4 g a day of THC-predominant cannabis (Russo et al., 2022), which contemporaneously likely exceeded 15% THC concentration. Episodes of CHS do not follow expected parameters of dose-response to toxic exposure such as concentration thresholds, however. Rather, anecdotal experience indicates that once CHS symptoms appear and become entrenched, even exposure to very low concentrations of such agents may induce an episode (Russo et al., 2022).

CHS is most commonly reported in North America, but figures on its prevalence are yet unclear, ranging from 350,000 in the USA to an extrapolated estimate of 2.75 million (Russo et al., 2022) based on data from one emergency department survey in New York City (Habboushe et al., 2018). CHS is often misdiagnosed, leading to prolonged delays before appropriate intervention and counseling and this accounts for repetitive clinic visits and unrevealing diagnostic tests with accompanying costs before diagnosis averaging more than $95,000 USD in 2012 (Perrotta et al., 2012). Certainly, recognition of the disorder has increased since its initial description, which has paralleled a marked escalation of THC concentrations in cannabis, along with more broad accessibility to those products, as well as synthetic alternatives.

CHS certainly can be considered as one of the few true contraindications to cannabis usage, but its implications are far more ominous given recent data from genomic testing (*vide infra*) (Russo et al., 2022) that indicate affected individuals are susceptible to numerous comorbidities, including addiction and other psychiatric sequelae, as well risks of future development of diabetes, coronary artery disease and dementia. Considering these facts, better diagnosis, treatment, and intervention are high priorities.

## 2 Schools of thought and controversies

### 2.1 Pathophysiology of CHS

Presumed aspects of pathophysiology of CHS have been well analyzed (DeVuono and Parker, 2020). In addition to the biphasic dose-response of nausea to THC, additional theories or constructs of its etiology include a downregulation of the CB_1_ receptor to the point at which THC acts contrarily as an antagonist, rather than a partial agonist (Sim-Selley et al., 2003), or activation of the hypothalamic-pituitary axis (HPA) with florid stress responses.

Hypothetical genetic factors in CHS were subsequently investigated (Russo et al., 2022), applying screening rigorous criteria, 205 patients were identified with an average daily intake of 4 g of high-THC cannabis. Twenty-eight of these returned genomic test kits and their results were compared with 54 cannabis users with equivalent cannabis usage rates, but without classic CHS symptoms. Results demonstrated that the CHS cohort lacked a single nucleotide polymorphism (snp) on the *CNR1* gene coding for the CB1 receptor, but displayed five other mutations statistically significantly distinguished from controls affecting *CYP2C9*, the gene coding the enzyme that metabolizes THC in the liver (*p* = 0.043), *TRPV1* the transient receptor potential vanilloid 1, for which capsaicin is a ligand (*p* = 0.015), the ATP-binding cassette transporter gene (*ABCA1*) (*p* = 0.012), and two genes affecting dopamine, *COMT* coding catechol-O-methyl transferase that catabolizes dopamine (*p* = 0.012), and *DRD2*, the gene for the dopamine-2 receptor (*p* = 0.031) (Russo et al., 2022). Considering the functions of these genes and their manifestations affecting nausea, gut motility, cannabinoid metabolism, and the abundant psychiatric manifestations of CHS (addiction, anxiety, compulsivity, et al.), it is illogical to pass off these findings as happenstance.

### 2.2 CHS triggers beyond Δ^9^-THC

Synthetic CB_1_ agonists have also been associated with the appearance of CHS symptomatology (Hopkins and Gilchrist, 2013). A male patient and former cannabis smoker with classic CHS presentation stopped usage for 6 months due to fears of urine testing for his employment, turning instead to synthetic cannabinoids of the JWH series (Denooz et al., 2013), which are high potency full CB_1_ agonists at the receptor, some displaying 100 times the potency of THC. These were developed as basic science tools that were never intended for human use. The patient relapsed into the CHS constellation secondary to utilization of these products. Synthetic cannabinoids were identified in the urine with no evidence of THC or its metabolites. His symptoms subsequently abated after 2 weeks of total abstinence.

A glut of cannabis production, particularly of cannabidiol (CBD) in the USA has generated a profusion of synthetically derived Δ^8^-THC, which also acts as weak partial agonist at the CB_1_ receptor (Bergeria et al., 2023), with potency estimated to be 63% of Δ^9^-THC (Hollister and Gillespie, 1973). A female patient developed CHS symptoms after 1 month of Δ^8^-THC usage in “gummies” taken as a sleep aide (Rosenthal et al., 2021). She subsequently responded to treatment with intravenous haloperidol and topical capsaicin.

CHS has also been implicated in a case report of a 6-year-old epileptic patient on Epidiolex^®^, a pharmaceutical with 98% pure CBD, and only traces of THC after its removal by partial centrifugal chromatography (Katz et al., 2023). However, this example is highly questionable, since THC exposure was extremely low, the patient was on a ketogenic diet with polypharmacy, and emesis episodes were only of 1–2-day duration five times in 6 months and recurred once after discontinuation of Epidiolex.

Numerous CHS patients have reported to us that once the syndrome has developed, and even after prolonged abstinence, they may trigger symptoms through exposure to certain scents, such as those of cannabis, which derive from its essential oil terpenoid components and not from the odorless phytocannabinoids (Russo, 2011). It is known from prior studies that apart from beta-caryophyllene, which is a selective CB_2_ agonist (Gertsch et al., 2008), none of the other common cannabis terpenoids display activity on the CB_1_ receptor (Santiago et al., 2019; Finlay et al., 2020), and should not account for resumption of CHS symptoms. The pathophysiology of this phenomenon may well be explained via classical conditioning theory, wherein the aroma of cannabis terpenoid components becomes psychologically associated with past cannabis use and induces resumption of CHS manifestations. This would be akin to mechanisms postulated to affect multiple chemical sensitivity patients (Siegel and Kreutzer, 1997). It remains to be determined if efforts at extinction therapy maneuvers will prove effective.

### 2.3 CHS, pesticides and neem oil

Experimental evidence reveals that when pesticides are employed in cannabis culture, up to 70% of their concentration may be recruited into its smoke (Sullivan et al., 2013). Use of such agents is rampant in unregulated jurisdictions, as revealed by a study of legal products in Washington State, USA (Russo, 2016), which documented pesticide contamination in 22/26 herbal cannabis and concentrate products (84.6%) with concentrations up to hundreds of thousands of parts per billion, including agents that are potential neurotoxins, carcinogens, acetylcholinesterase inhibitors, reproductive toxins and endocrine disruptors. While the full implications of chronic exposure to such agents remain to be determined, their acute effects are quite distinct from those manifested in CHS; Anecdotal reports from CHS patients and analytical laboratories maintain that the disorder still occurs from use of organic and pesticide-free cannabis material.

**FIGURE 1:**
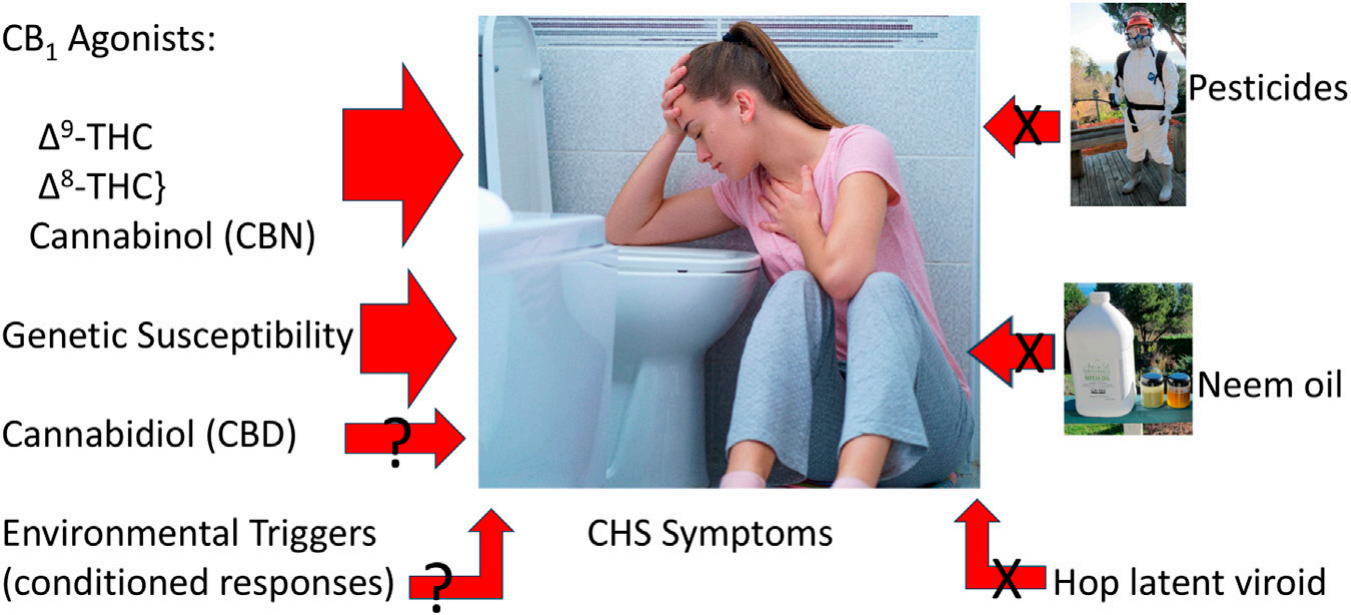
Triggers to cannabinoid hyperemesis syndrome (Center image licensed from https://123rf.com, also utilized at (CReDO Science, 2024), neem oil photo by EBR, pesticide spray photo by Kay Frey, with permission).

Other claims have implicated CHS due to use of neem oil from the seed of *Azadirachta indica*, or its primary insecticidal agent, azadirachtin. Reported toxicological sequelae from these agents are notably thin. In India, burning neem oil in kerosene effectively deters mosquito bites (Sharma and Ansari, 1994), but kerosene burns at 182°C–239°C, whereas the boiling point of azadirachtin is 792°C, in contrast to the temperature of the burning end of a cannabis joint has been estimated at 700°C at its height (PubChem, 2024) Thus, cannabis smoking or vaporization of materials with residual azadirachtin would consist of vapor, rather than any unique or potentially more toxic byproducts Figure 1.

Based on chronic administration of azadirachtin to rats, a non-toxic dose for human consumption was calculated to be 0.014 g/kg body weight (Boeke et al., 2004) [or 0.98 g for a 70 kg human]. The authors stated, “---the toxic effects of neem oil are unlikely to be caused by its azadirachtin content.” p. 35. The estimated safe daily dose for unprocessed neem material was 0.25 mg/kg BW [or 17.5 mg for a 70 kg human].

Accidental ingestion of neem oil by a 5-year-old boy (amount unspecified) resulted in status epilepticus and cardiac arrest with hypoxic brain damage and subsequent choreoathetoid movements after 2 months (Dhongade et al., 2008). In another instance, a 35-year-old female intentionally ingested an azadirachtin preparation (estimated 2.5 g), producing drowsiness and leukocytosis, but no renal or hepatic complications. There were no long-term sequelae (Iyyadurai et al., 2010). Case reports from SE Asia of neem ingestion in children note hepatic lipid deposition, seizures, and sedation.

While neem oil and azadirachtin are popular preventative organic pesticide products for cannabis cultivation, they should only be applied pre-flowering. They break down entirely after 3–4 days. Concentrations encountered in cannabis smoking or vaporization would be expected to be exceptionally low and unlikely to produce symptoms in adult humans. Ultimately, observed toxicity of neem oil and azadirachtin do not match those of CHS.

### 2.4 CHS and plant viruses

It has been suggested in some quarters without corroborative evidence that CHS could be attributed to the profusion of cannabis cultivars infected with hop latent viroid (Dorantes, 2021). This allegation smacks of conspiracy theory (Russo, 2022), because no plant virus has ever been documented to make the great leap to infect humans (Balique et al., 2015).

### 2.5 Possible CHS variation

It has been proposed that gradations of CHS might occur clinically. Two case reports detailed patients with complicated histories involving celiac disease, opioid usage and other factors who suffered symptoms of nausea and lower gastrointestinal problems without vomiting or alleviation by hot water (Sulak and Theisen, 2019). Both were able to tolerate intermittent cannabis usage in low concentrations, but with relapsing symptoms after any dose escalation.

### 2.6 Cannabinoid hyperemesis syndrome vs. cyclic vomiting syndrome (CVS)

A serious misconception has recently been conveyed in the AGA Clinical Practice Update on cannabinoid hyperemesis syndrome (Rubio-Tapia et al., 2024), which states, “CHS is a subtype of cyclic vomiting syndrome (CVS)----.” While it may seem nosologically convenient to “lump” these syndromes together, it is scientifically untenable, because their pathophysiology and treatments are entirely different, and that they can be distinguished through careful history-taking and genomic testing (Russo et al., 2022).

Cyclic vomiting syndrome is a *forme fruste* of migraine, most often appearing in infancy or childhood with episodic vomiting in the absence of headache symptoms but with a strong family history of same. Only subsequently may the child or adult develop more classic hemicranial pain, visual disturbance, or other accoutrements of migraine. CVS was demonstrated to be associated with single nucleotide polymorphisms (snp) consisting of AG and GG genotypes of the *CNR1*, the gene encoding the CB_1_ receptor where THC exerts its most prominent effects on brain and gut (Wasilewski et al., 2017). This snp was notably absent in the cohort of CHS patients who underwent genomic testing (Russo et al., 2022). Of additional distinction, adult CVS sufferers have turned to treatment with cannabis with reported benefit (Siddiqui et al., 2020), creating some confusion for clinicians evaluating patients with episodic vomiting attacks, unless more in-depth interviewing is applied, a notable challenge given the demands of contemporary medical practice. Whereas CHS was notably under-diagnosed or missed entirely in the past, the proverbial tide has turned such that currently in North America, any young person presenting with repetitive vomiting who admits to cannabis usage in any context may be misdiagnosed on the spot as having CHS by a clinician in too much of a hurry such that fine diagnostic distinctions and historical details are not considered. Applying proper interviewing techniques coupled with a non-invasive oral swab for genomic assessment may obviate needless clinical detours, and avoid pitfalls of conflating CHS and CVS, thus avoiding the pitfall of ascertainment bias.

## 3 Current Research Gaps.

### 3.1 Can cannabis be a treatment for CHS instead of its cause?

Among the features of CHS are the great resistance that most of its sufferers display toward the concept that cannabis is making them sick. Everyone knows cannabis and Marinol^®^ (dronabinol, synthetic THC) are helpful in allaying nausea and vomiting attendant to cancer chemotherapy. This prevalent attitude is in part responsible for the astounding relapse rate CHS patients suffer as they resume and escalate their dosages: 79% of 204 CHS respondents indicated they returned to cannabis usage after diagnosis (Russo et al., 2022) (Supplemental Material), despite tremendous pain, suffering and associated costs. The only proven path to improvement is abstinence from cannabis/CB_1_ agonists.

Some CHS patients have attempted substitution with cannabidiol (CBD) products but with no success. CBD sourced from herbal cannabis is rarely free of THC, because cannabidiolic acid synthase, the enzyme that is responsible for producing cannabidiolic acid from cannabigerolic acid, also produces a small fraction of tetrahydrocannabinolic acid (Onofri et al., 2015), which with aging or exposure to heat or light decarboxylates to THC. Additionally, quality control is not exemplary in the poorly regulated cannabis market, and labelling is frequently highly inaccurate (Vandrey et al., 2015). As an anti-emetic in its own right (Parker et al., 2011) and a negative allosteric modulator of CB_1_, CBD might be expected to be of benefit as a CHS treatment, if it were pure. An additional negative factor would be the ability of CBD to inhibit weakly *in vitro* fatty acid amidohydrolase (FAAH) (Bisogno et al., 2001), the enzyme that catabolizes the endocannabinoid, anandamide. This may increase endocannabinoid tone and be a possible exacerbating factor in CHS susceptibility.

Cannabigerol (CBG) also inhibits FAAH (Bisogno et al., 2001) but has been reported in surveys to alleviate gastrointestinal symptoms including inflammatory bowel diseases (Russo et al., 2021). Prior testing of CBG (Rock et al., 2011) failed to demonstrate anti-emetic effects.

Δ^8^-THC, as noted above, a less potent analogue of Δ^9^-THC (Pertwee and Cascio, 2014), is not a suitable alternative for CHS patients. Similarly, cannabinol (CBN), a popular agent for sleep disturbance despite negative scientific support (Corroon, 2021), is also a weak partial CB_1_ agonist (Ki > 100 nM) (Pertwee and Cascio, 2014), that can be expected to be a putative trigger of symptoms.

Tetrahydrocannabivarin (THCV) has an unusual profile as a neutral antagonist of CB_1_ at low doses, but an agonist at much higher doses (Thomas et al., 2005). Although selective breeding has produced plants with 93% of total cannabinoid content as THCV (de Meijer and Hammond, 2016), residual THC content remains the rule, as THCA synthase produces both the pentyl and propyl molecules in the cannabis plant.

Tetrahydrocannabinolic acid (THCA) is anti-emetic in laboratory animals (Rock et al., 2020), but is an unstable molecule that decarboxylates to THC, and is likely problematic. In contrast, cannabidiolic acid (CBDA) is strongly anti-emetic via stimulation of serotonin 1A receptor, much more potently than CBD (Bolognini et al., 2013) and might be an interesting agent to test in CHS patients, especially if stabilized as a methoxy-CBDA pro-drug (Rock et al., 2021).

Although one might consider the use of more powerful CB_1_ antagonists for CHS, these are unlikely to be of benefit. SR141716-A was previously marketed briefly in Europe as Rimonabant^®^, but this powerful inverse agonist of CB_1_ lowered endocannabinoid tone, but also produced nausea, and its propensity to increase anxiety, depression and suicidality led to its removal from the market (McPartland et al., 2015).

### 3.2 CHS psychiatric, medical implications and comorbidities

Whereas the five mutations in CHS patients that were statistically significantly different from their incidence in chronic cannabis users without such symptoms explain much of the underlying pathophysiology of gastrointestinal motility issues, nausea, vomiting and the idiosyncratic benefits of hot water and topical capsaicin (Russo et al., 2022; Russo et al., 2021), they raise many additional red flags for this population with respect to additional morbidities. Mutations on catechol-O-methyl transferase (*COMT*) and *DRD2* dopamine genes portend addiction problems with alcohol and other drugs beyond cannabis, as well as susceptibility to chronic pain, depression, anxiety and psychosis (Nyman et al., 2011; Pap et al., 2012; Stadlin et al., 2014). If that were not sufficiently ominous, observed mutations on the ATP-binding cassette transporter (*ABCA1*) could increase risks of future abnormal protein deposition in the brain, dementia, coronary artery disease and Type II diabetes (Feher et al., 2018). Thus, early diagnosis with appropriate genetic and health counseling are high priorities upon diagnosis. The commonly encountered resistance of CHS patients makes this recommendation notably challenging.

## 4 Potential future developments

### 4.1 Diagnostic and psychometric testing toward a better understanding of CHS

The issues highlighted above support the advisability of more applied research on CHS patients, especially additional investigation of genomic testing to confirm prior results, seek additional mutations of interest, and ascertain if it represents a valid diagnostic tool. We also recommend that psychometric testing be examined to assess comorbidities of anxiety, depression and addiction potential that are suggested susceptibilities from prior study (Russo et al., 2022). Given logistical challenges in recruitment and retention, such an effort will require funding sources to mount multicenter studies.

### 4.2 Metabolomic testing of CHS

A fascinating study has recently demonstrated highly specific metabolic changes accompanying symptomatic improvement of autistic patients with cannabis-based therapies (Quillet et al., 2023). Hypothetically, analogous metabolomic testing of CHS patients may provide additional elucidation of its pathophysiology and highlight possible future approaches to its treatment. Inasmuch as total abstinence from cannabis is currently the only recognized successful intervention in CHS patients, future treatment should focus on symptom alleviation, rather than finding agents that will allow patients to continue cannabis usage.

## 5 Discussion/conclusion

Cannabinoid hyperemesis syndrome has developed from a rare and often misdiagnosed curiosity into a common presenting complaint at emergency departments, particularly in North America. The rest of the world is not far behind given the profusion of high potency herbal cannabis, its extracts and synthetic cannabinoids, the latter arising as a byproduct of prohibition. It is increasingly clear that CHS is an emerging public health risk and one that requires additional research, proper funding, and efforts at preventative education for one of the few true contraindications to cannabis usage.
